# Anti-Vivisectionists and Their Mistakes

**Published:** 1892-12-03

**Authors:** 


### EDITOR'S LETTER BOX.

### ANTIVIVISECTIONISTS AND THEIR MISTAKES.

Miss Ethel M. Warner writes as follows: The statements made by the antivivisectionists are usually incorrect; and in spite of Bishop Barry's parting advice to members of the Antivivisection Society to be very sure of their facts in future before making accusations, another false statement finds its place in Mr. Lawson Tait's letter to The Hospital for November 19th. Allow me, as having had charge of some of Mr. Victor Horsley's first operation cases for brain tumour, to correct Mr. Lawson Tait's statement that "Mr. Horsley's celebrated successful case died at about the end of a month." Mr. Victor was the first who removed a brain tumour with the ultimate recovery of his patients. Your correspondent, I presume, is not aware that the first operation for brain tumour was performed in London by Mr. Rickman Godlee on November 25th, 1884, but the patient died from some intercurrent disease ten days afterwards. Mr. Godlee read a paper on the subject before a meeting of the "Royal Medical and Chirurgical Society," to which Professor Macewen came from Scotland to be present, when he stated the results of some operations he had performed since 1876, but amongst which the removal of a brain tumour was not included; he also stated that "the researches of Dr. Hughling Jackson, Dr. Ferrier, and others had greatly assisted" him, which contradicts your correspondent's remarks. The second operation for brain tumour was performed by Professor Victor Horsley, at the National Hospital, Queen Square, the patient recovering from the operation, and being cured of his malady. Equally good results followed from the direct guidance of experiment. I have in my possession at the present time a letter written to me by a patient six months after Mr. Horsley removed a tumour from his brain in 1886. Your correspondent may find it advisable to study a book which will shortly be published by the Association for the Advancement of Medicine by Research, before making further statements which are incorrect.

